# Supplementary material for: The Role of MER Processing Pipelines for STN Functional Identification During DBS Surgery: A Feature-Based Machine Learning Approach
Source: Bioengineering (Basel). 2025 Nov 26;12(12):1300. doi: 10.3390/bioengineering12121300 (PMC12729932; doi:10.3390/bioengineering12121300)
Supplement: Supplementary file 1 [file bioengineering-12-01300-s001.zip › bioengineering-3949511-supplementary.pdf]

Supplementary document for: The role of MER processing pipelines for STN functional identification during DBS surgery: a feature-based machine learning approach

Vincenzo Levi, Stefania Coelli, Chiara Gorlini, Federica Forzanini, Sara Rinaldo, Nico Golfrè Andreasi, Luigi Romito, Roberto Eleopra and Anna Maria Bianchi

Additional statistical results representation: Figure S1, Figure S2, Figure S3 and Figure S4

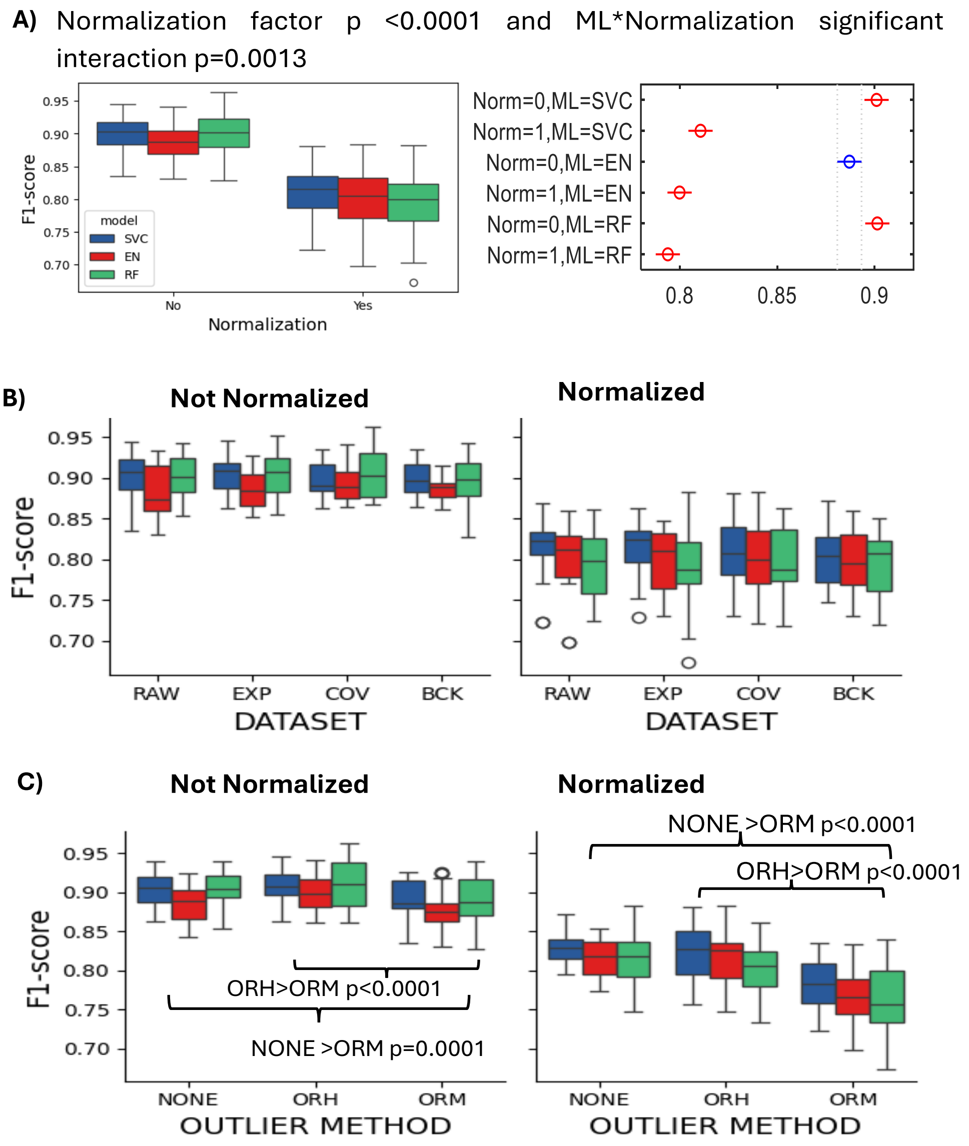

Figure S1. F1-score values distributions in function of pipeline components of interest. A) F1-score values for each ML models (SVC: blue, EN: red, RF: green) applied to the normalized and not normalized feature datasets (left) and (right) the analysis of the significant main factors' interaction (NORMALIZATION*ML). B) F1-score values, in function of the DATASET obtained applying different artifacts rejection approaches for each ML model for the classification of normalized and not-normalized

features. The 'DATASET' factor was not significant while a significant ML factor effect was found in both cases (Not Normalized p<0.0001, Normalized p=0.004). C) F1-score values, in function of the OUTLIER management methods applied for each ML model for the classification of normalized and not normalized features. Brackets identify significant differences related to the effect of the OUTLIER method applied.

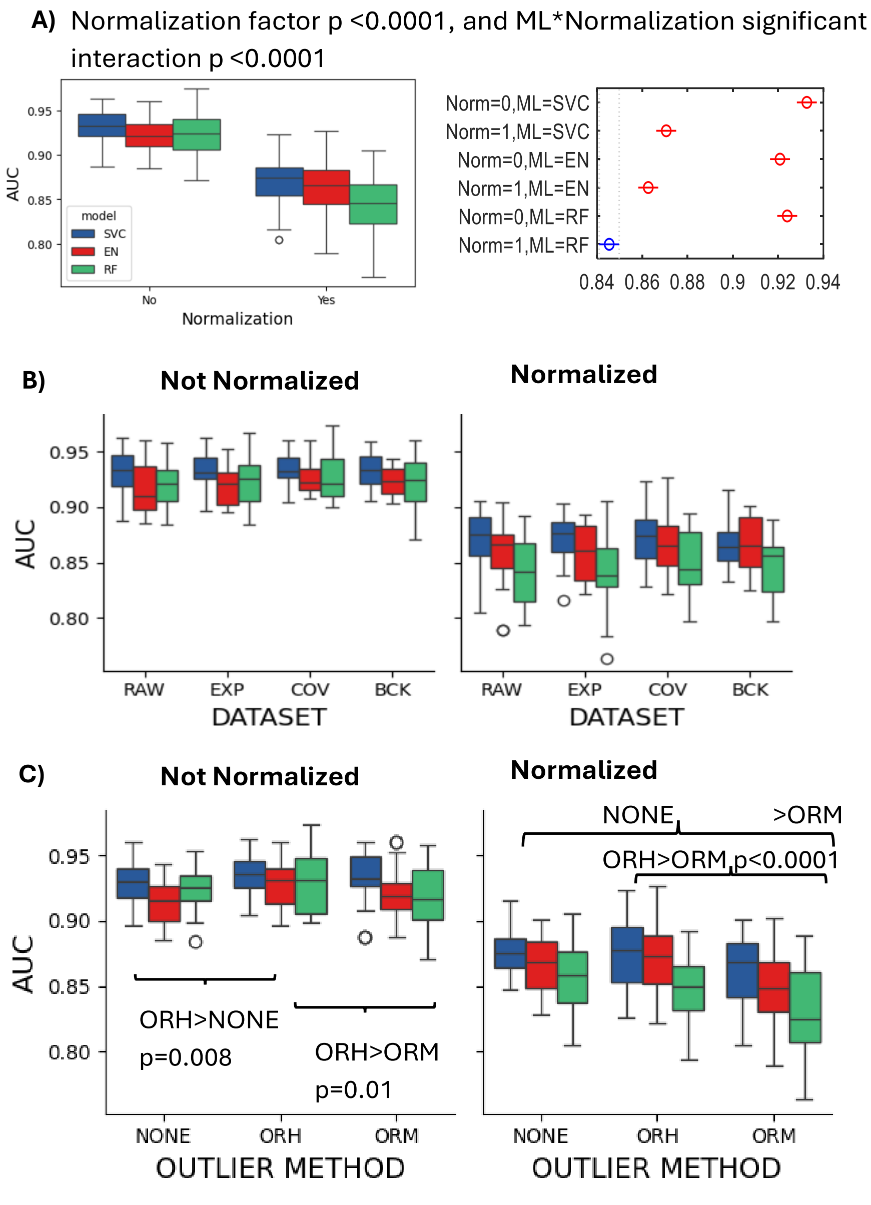

Figure S2. AUC values distributions in function of pipeline components of interest. A) AUC values for each ML models (SVC: blue, EN: red, RF: green) applied to the normalized and not normalized feature datasets (left) and (right) the analysis of the significant main factors' interaction (NORMALIZATION*ML). B) AUC values, in function of the DATASET obtained applying different artifacts rejection approaches for each ML model for the classification of normalized and not-normalized features. The 'DATASET' factor was not significant while a significant ML factor effect was found in both cases (p<0.0001). C) AUC values, in function of the OUTLIER management methods applied for each ML model for the classification of normalized and not-normalized features. Brackets identify significant differences related to the effect of the OUTLIER method applied.

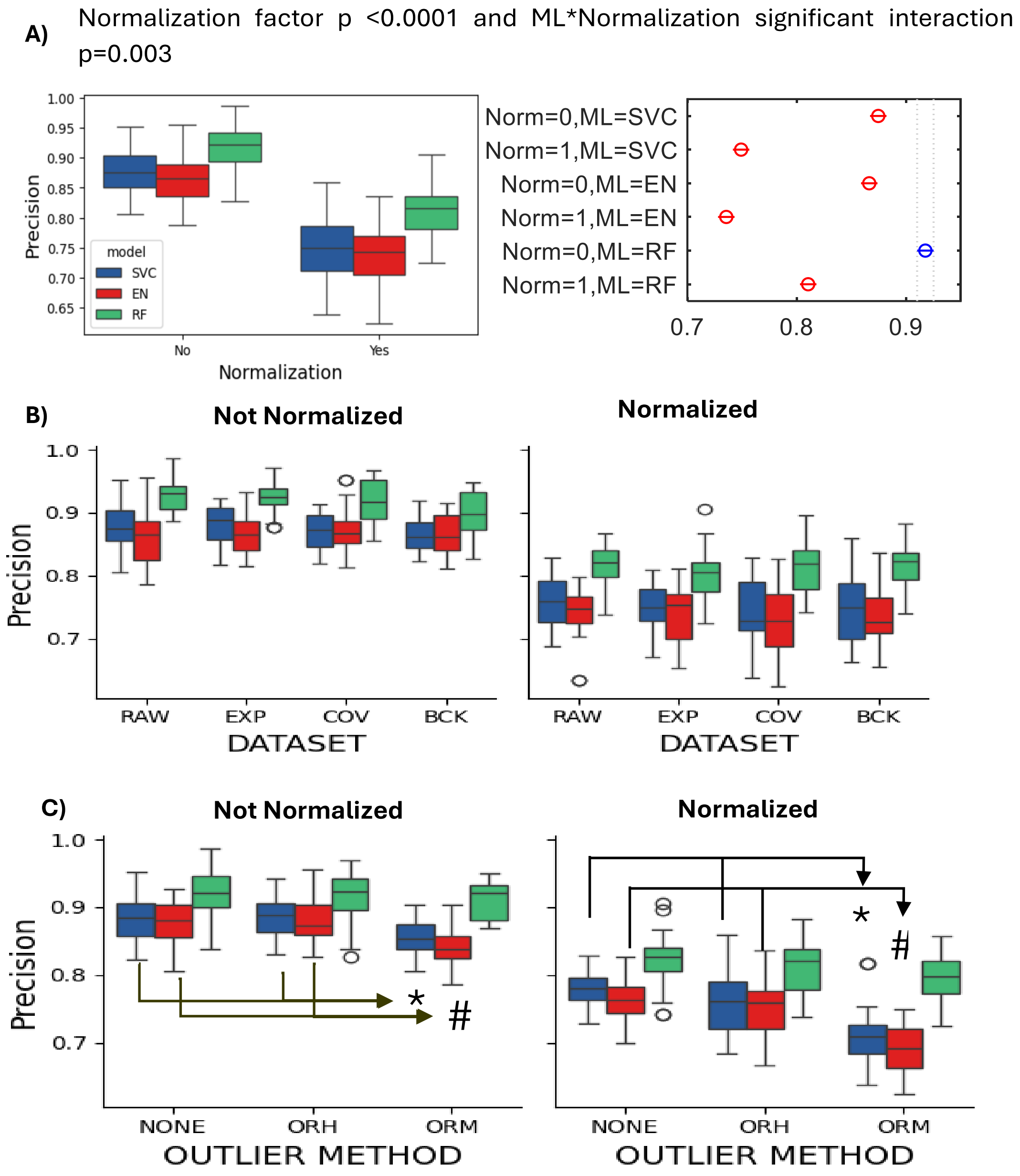

Figure S3. Precision values distributions in function of pipeline components of interest. A) Precision values for each ML models (SVC: blue, EN: red, RF: green) applied to the normalized and not normalized feature datasets (left) and (right) the analysis of the significant main factors' interaction (NORMALIZATION*ML). B) Precision values, in function of the DATASET obtained applying different artifacts rejection approaches for each ML model for the classification of normalized and not-normalized features. The 'DATASET' factor was not significant while a significant ML factor effect was found in both cases ($p<0.0001$). C) Precision values, in function of the OUTLIER management methods applied for each ML model for the classification of normalized and not normalized features. In both cases the interaction ML*OUTLIER was significant (normalized: $p=0.015$, not normalized: $p=0.0001$). # identifies the lowest value in the case of EN model and * identifies the lowest Precision when SVC was applied. In both normalized and not normalized case ORM decreases classification precision.

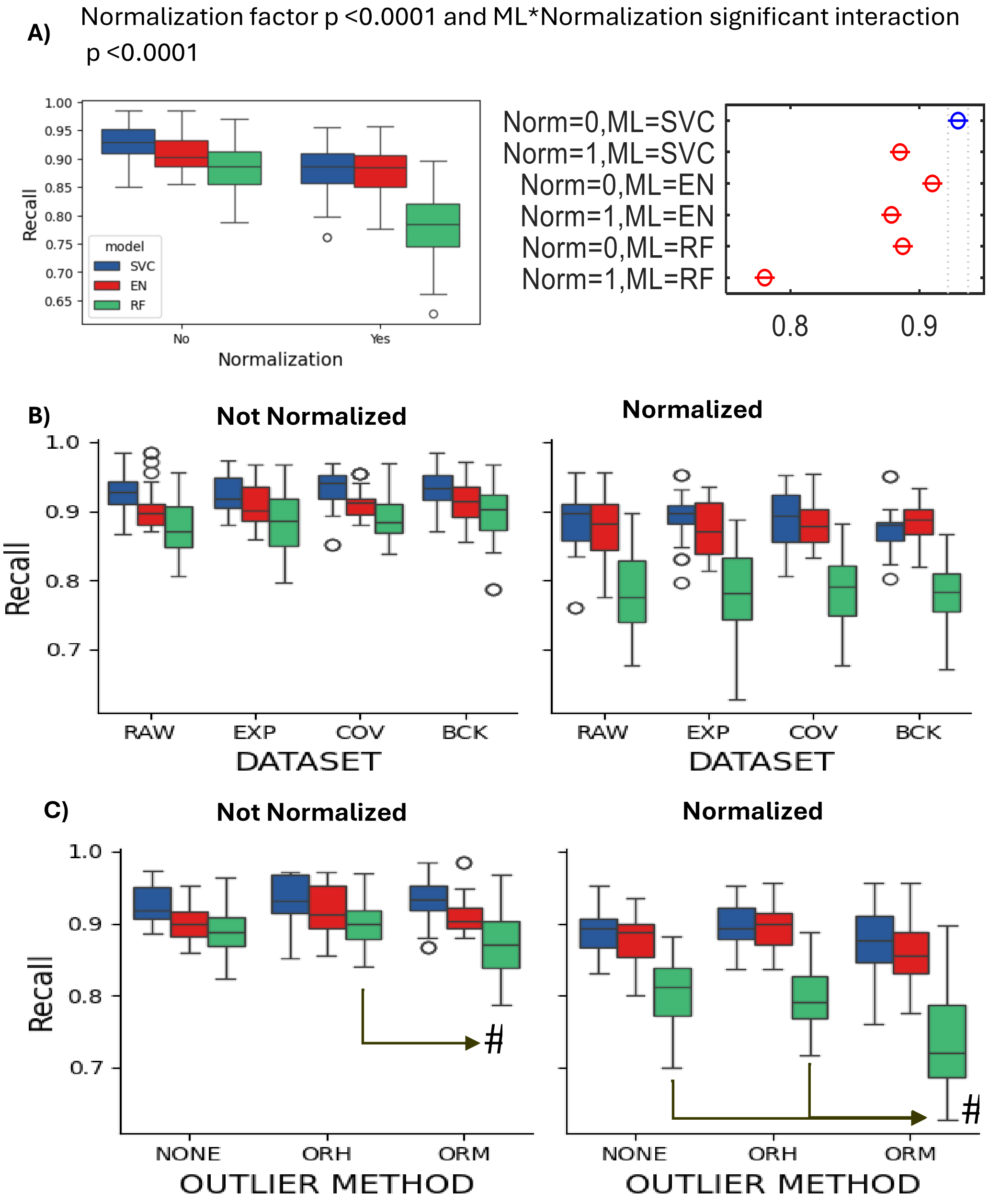

Figure S4. Recall values distributions in function of pipeline components of interest. A) Recall values for each ML models (SVC: blue, EN: red, RF: green) applied to the normalized and not normalized feature datasets (left) and (right) the analysis of the significant main factors' interaction (NORMALIZATION*ML). B) Recall values, in function of the DATASET obtained applying different artifacts rejection approaches for each ML model for the classification of normalized and not-normalized features. The 'DATASET' factor was not significant while a significant ML factor effect was found in both cases ($p<0.0001$). C) Recall values, in function of the OUTLIER management methods applied for each ML model for the classification of normalized and not-normalized features. In both cases the interaction ML*OUTLIER was significant (normalized: $p=0.007$, not normalized: $p=0.0008$). # identifies the lowest value in the case of RF model. In both normalized and not normalized case ORM decreases classification recall.
